# Hospital clinicians’ psychosocial well-being during the COVID-19 pandemic: longitudinal study

**DOI:** 10.1093/occmed/kqac003

**Published:** 2022-03-19

**Authors:** Karen Wynter, Sara Holton, Melody Trueman, Suellen Bruce, Sue Sweeney, Shane Crowe, Adrian Dabscheck, Paul Eleftheriou, Sarah Booth, Danielle Hitch, Catherine M Said, Kimberley J Haines, Bodil Rasmussen

**Affiliations:** 1 School of Nursing and Midwifery, Faculty of Health, Deakin University, Geelong, 3220, Australia; 2 The Centre for Quality and Patient Safety Research in the Institute of Health Transformation, Deakin University, Western Health Partnership, St Albans, 3021, Australia; 3 Nursing and Midwifery, Western Health, St Albans, 3021, Australia; 4 People, Culture and Communications, Western Health, Footscray, 3021, Australia; 5 Medical Services, Western Health, St Albans, 3021, Australia; 6 Allied Health, Western Health, St Albans, 3021, Australia; 7 Physiotherapy, Melbourne School of Health Sciences, The University of Melbourne, Parkville, 3010, Australia; 8 Australian Institute of Musculoskeletal Sciences, St Albans, 3021, Australia; 9 Faculty of Health Sciences, University of Southern Denmark and Steno Diabetes Center, Odense, 5230, Denmark; 10 Faculty of Health and Medical Sciences, University of Copenhagen, Copenhagen, 1165, Denmark

**Keywords:** Hospital clinicians, psychosocial, psychological health, workplace stress

## Abstract

**Background:**

Hospital clinicians report poor psychosocial well-being during the COVID-19 pandemic. Few studies have reported data at more than one time point.

**Aims:**

To compare psychosocial well-being among hospital clinicians at two different time points during the COVID-19 pandemic in 2020.

**Methods:**

Participants included doctors, nurses, midwives and allied health clinicians at a multi-site, public health service in Melbourne, Australia. Data were collected via two cross-sectional, online surveys: May to June (wave 1; *n* = 638) and October to December 2020 (wave 2; *n* = 358). The Depression, Anxiety and Stress Scale (DASS-21) assessed psychological well-being in the past week. Investigator-devised questions assessed COVID-19 concerns and perceived work impacts. General linear models were used to assess impact of wave on psychological distress.

**Results:**

There were no significant demographic differences between the two groups. Both positive (e.g. learning experience) and negative (e.g. risk of getting COVID-19) impacts were reported. In both waves, staff were most concerned about health risks to family members. Wave 2 respondents were significantly more likely than wave 1 respondents to indicate concerns about colleagues having COVID-19, increased workloads, leave cancellation and increased conflict at work (all *P* < 0.001). Adjusting for sex, age, self-rated health and discipline group, depression, anxiety and stress scores were significantly higher for respondents in the second than the first wave (all *P* < 0.001).

**Conclusions:**

Psychological well-being of hospital clinicians was significantly worse during the second wave of the COVID-19 pandemic than the first. Sustained occupational and psychosocial support is recommended even when immediate COVID-19 concerns and impacts resolve.

**Key learning points**

**What is already known:**

- Poor psychological well-being has been found to be common among hospital clinicians during the COVID-19 pandemic.
- Clinicians have reported being concerned about their own exposure to COVID-19 and infecting family members.
- There is currently little evidence about changes in psychosocial well-being among hospital clinicians over time, as the pandemic progresses.

**What this study adds:**

- Clinicians assessed during a second, more intense wave of COVID-19 reported significantly higher scores on all subscales of the DASS-21, compared to staff at the same health service during the first wave five months earlier.
- Compared with doctors, nurses and midwives reported significantly more anxiety at both time points.
- Clinicians were significantly more concerned about colleagues getting COVID-19 in the second wave compared to the first wave.
- Clinicians were significantly more likely to report increased workloads, cancelled annual leave, increased conflict and decreased cooperation among staff during the second wave compared to the first wave.

**Impact on practice or policy:**

- As the COVID-19 pandemic continues, clinicians will benefit from increased and ongoing organisational and psychological support.
- For future pandemics and other adverse events, manageable workloads and permitted leave periods for clinicians should be facilitated by careful workforce planning.
- Clinicians’ education should equip them to manage fears and concerns during a pandemic; these could include specific self-care, building resilience and healthy coping skills, and developing efficient and effective teamwork.

## Introduction

There is already clear evidence from several reviews and meta-analyses [1-4] that a substantial proportion of healthcare workers have experienced clinically significant levels of psychological distress during the COVID-19 pandemic. In one recent meta-analysis [1], the pooled prevalence of anxiety among healthcare workers was 22% (95% CI: 19–25%) and depression 23% (95% CI: 21–26%).

A limitation of almost all studies reporting psychological distress or well-being among healthcare workers is that these studies have been cross-sectional in design [1-6]. Few studies have collected longitudinal data demonstrating the ongoing psychosocial well-being of hospital clinicians during successive ‘waves’ of the COVID-19 pandemic. A limited number of studies in China, Japan and Canada have reported data from healthcare workers at more than one time point. These studies compare clinicians’ mental health during the COVID-19 pandemic with before [7] or after [8, 9] such an outbreak; anxiety [7-9], depression [7-9], post-traumatic stress [9] and insomnia [7] were reported to be significantly higher during the pandemic ‘waves’ than at other times.

There are currently few studies which have demonstrated changes in psychological well-being among healthcare worker well-being over time during multiple waves of the COVID-19 pandemic. One study in China compared responses as the pandemic progressed and found that while anxiety about the COVID-19 pandemic (assessed by individual items rather than a standardised measure) lessened, respondents’ sleep quality did not improve [10]; this study was limited by a very short timeframe for the comparison of data (2 weeks) [10]. A Japanese study tracked healthcare workers’ psychological distress from the early phase of the first wave (March 2020) until the early phase of third outbreak (November 2020) of the COVID-19 pandemic. Being a healthcare worker was associated with a significant increase in psychological distress (assessed using a brief job stress inventory) over time [11]. However, of the 111 healthcare workers included in this study, only 19 worked in clinical settings [11] Thus, limited longitudinal data about psychological distress or well-being among clinical, hospital-based healthcare workers are available. Without such data it is difficult to determine whether healthcare workers’ well-being changes between subsequent waves of the COVID-19 pandemic.

Studies assessing healthcare workers’ specific psychosocial concerns and perceived impacts of pandemics such as COVID-19 have mostly been cross-sectional [2, 4, 5]. Most commonly, during the COVID-19 pandemic healthcare workers were concerned about being exposed to or infected with COVID-19, and infecting others. A study in the UK revealed that COVID-19 demands, along with hours worked, were related to higher mental health strain among emergency medicine personnel [12]. No studies have assessed changes in pandemic-related concerns and perceived impacts over time.

The first aim of this study was to compare, across two time points during 2020, hospital clinicians’ COVID-19 related concerns and perceived work impacts. The second aim was to investigate the impact of ‘wave’ (time point) and discipline group (nurses and midwives, allied health clinicians, medical staff) on clinicians’ levels of depression, anxiety and stress.

## Methods

The study design involved comparison of data from two independent groups collected 5 months apart, and simultaneous testing of the effect of wave and discipline group on DASS-21 subscale scores using general linear models (GLM).

Melbourne’s first ‘lockdown’ was implemented from 24 March 2020 until 11 May 2020 [13, 14]. During this lockdown, Melbourne was in ‘stage 3’ restrictions which included the closure of non-essential services and schools, and physical distancing. Melbourne experienced a second wave of the pandemic with an increased number of COVID-19 cases and deaths, and as a result, another lockdown was implemented from 7 July 2020 until 28 October 2020

Western Health is located in metropolitan Melbourne, Australia. It includes three acute hospitals, a day hospital, a transition care programme and a drug and alcohol service, and provides acute tertiary services, subacute care, specialist ambulatory clinics and community health services. Western Health is the main healthcare provider for the population of western Melbourne (where 41% of cases have occurred [15]). It provided inpatient care for over 400 patients with COVID during 2020, including 65 who were admitted to ICU, and 72 who died in hospital (personal communication [16]).

Convenience sampling was used at each time point. All hospital clinicians (approximately 4350: 3000 nurses and midwives, 329 allied health clinicians, and 1200 doctors) were invited to participate in an online survey towards the end of each wave in Melbourne: May–June 2020 (wave 1), and October–December 2020 (wave 2). The surveys were available in Qualtrics [17], an online survey platform. At each time point, all doctors, nurses, midwives, and allied health clinicians employed at the health service were sent an e-mail inviting them to participate. One reminder was sent, a few weeks after each initial invitation. Emails included a link to the anonymous survey and a participant information statement. Consent was implied by participants completing and submitting the survey

The survey content was developed based on existing studies assessing psychological well-being among healthcare workers during previous pandemics and epidemics (SARS: Severe Acute Respiratory Syndrome; MERS-CoV: Middle East Respiratory Syndrome Coronavirus) [18-23], and the clinical experience of the research team.

The survey included mostly fixed-response questions and assessed four domains. Firstly, respondents were asked some questions about themselves including demographic characteristics and years of clinical experience. Secondly, respondents’ health status was assessed, including their self-rated general health status (excellent, very good, good, fair or poor) and whether they had been exposed to COVID-19. Thirdly, a section on COVID-19 concerns and work impacts: In the first six items, participants were asked to indicate how concerned they were (from ‘not concerned’ to ‘extremely concerned’, on a 5-point Likert scale) about various potential impacts of COVID-19 on their own health and that of their family. In the next 15 items, participants indicated their level of agreement (from ‘strongly disagree’ to ‘strongly agree’, on a 5-point Likert scale) with statements about potential impacts of COVID-19 on their work. Finally, symptoms of depression, anxiety and stress during the past week were assessed using the widely used 21-item Depression, Anxiety and Stress Scale (DASS-21) [24]. Scores on each subscale range from 0 (no distress) to 21 (most distressed). Clinical cut-off points for each subscale have been established (Table 3) [24]. The survey was the same for each wave. To allow anonymous surveys but enable longitudinal matching of waves 1 and 2 surveys, in each survey respondents were asked to create a unique identification code using a specific combination of letters and numbers from their personal details (e.g. name and date of birth). Precise and identical instructions for generating this code in both surveys were provided to respondents. Examples were provided, and participants were informed that the format of this code was required so that they could remember it if they decided to participate in a subsequent survey, in order to match their data from both surveys.

In the first wave for the whole sample, the mean (SD) for the DASS Depression Scale was 3.08 (3.87) [25]. For an independent samples *t*-test comparing depression scores in waves 1 and 2, to yield at least a small effect size of Cohen’s *d* = 0.2, with 80% power and α = 0.05, the minimum sample size required in each group is 237. This sample size is sufficient for multivariate analysis.

IBM SPSS Statistics version 26 was used to analyse data. Wave 1 data have been reported in detail [25, 26].

To compare responses from the waves 1 and 2 surveys, firstly data were matched using the unique identification codes generated by respondents. This resulted in only 57 paired responses from respondents who completed both surveys and responded to both requests to generate a personal code. Accordingly, the data from the two surveys were treated as independent samples.

The distributions of respondents’ demographic and health characteristics in waves 1 and 2 were compared to identify any significant differences. Chi-square tests (with the continuity correction) were used to compare categorical variables. All continuous distributions were significantly non-normal; therefore, groups were compared groups using Mann-Whitney *U* tests. Means are provided for ease of interpretation but were not used for the analysis.

Responses to items about COVID-19 concerns and impacts were compared between waves 1 and 2 samples, for the combined sample and for each discipline group separately. In Australia, nurses and midwives are considered as one discipline group in the health workforce [27]. Nurses’ and midwives’ roles both involve direct, sustained patient contact. Therefore, the risk of infection may be considered similar for nurses and midwives, compared with medical and allied health staff. For these reasons, responses from nurses and midwives were combined. Likert-scale item responses were collapsed into two categories: ‘not concerned’ or ‘extremely/very concerned’; and ‘strongly disagree/disagree/neither disagree nor agree’ or ‘strongly agree/agree’. Chi-square tests (with the continuity correction) were applied to complete cases; thus, the total number of cases differs for each analysis.

DASS-21 subscale scores were calculated as per the manual for this instrument [24]. For each subscale of the DASS-21, in the case of one missing item, subscale scores as the mean of the remaining items was calculated for the relevant subscale. In the case of two or more missing items, subscale scores were not calculated. Cronbach’s α was calculated as an indication of internal consistency for each subscale. The proportion of respondents scoring in clinical ranges was calculated [24]. For the combined sample, as well as within each discipline group separately, DASS-21 subscale scores were compared, as were the proportions of respondents who scored in the moderate to extremely severe range of each subscale.

To investigate the impact of wave (time point) and discipline group on clinicians’ levels of depression, anxiety and stress, general linear models were used. Wave and discipline group were entered as fixed factors, and the analysis was adjusted for participants’ sex and age and, based on associations identified in the first wave [25], self-rated general health. Models were run with and without interaction terms between discipline group and wave. None of the interaction terms was significant. Therefore, the models without interaction terms are reported. Partial *η*^2^ is reported as an indicator of effect size.

Ethics approval was granted by the Western Health Low Risk Human Ethics Panel (HREC/20/WH/62913, 5 May 2020) and the Deakin University Human Research Ethics Committee (2020-321, 30 September 2020).

## Results

The wave 1 survey was completed by 668 respondents (391 nurses and midwives, 139 allied health clinicians and 138 doctors). The wave 2 survey was completed by 358 respondents (184 nurses and midwives, 74 allied health clinicians and 100 doctors). If all 4530 clinical staff at the study health service accessed their email invitations, this would represent a response rate of 15% and 8% for each survey: 13% and 6% for nurses and midwives, 42% and 23% for allied health clinicians and 12% and 8% for doctors.

There were no statistically significant differences in demographic characteristics (Table 1). Self-rated general health status declined significantly (*P* < 0.05) between waves 1 and 2 overall samples but not for any of the discipline groups. A significantly higher proportion of respondents overall and in each discipline group had been exposed to people with COVID-19 in wave 2 compared with wave 1 (*P <* 0.001; Table 1).

**Table 1.** Respondents’ sociodemographic characteristics: *n* (%) unless otherwise specified

	Wave 1				Wave 2			
Characteristic	Nurses/midwives	Allied health	Doctors	Total	Nurses/midwives	Allied health	Doctors	Total
Sex	*n* = 375¥	*n* = 135	*n* = 128	*n* = 638	*n* = 184	*n* = 74	*n* = 100	*n* = 358
Female	345 (92)	121 (90)	76 (59)	542 (85)	169 (92)	64 (87)	59 (59)	292 (82)
Male	27 (7)	12 (9)	50 (39)	89 (14)	15 (8)	10 (13)	41 (41)	66 (18)
Other/prefer not to say	3 (1)	2 (1)	2 (2)	7 (1)	0 (0)	0 (0)	0 (0)	0 (0)
Age	*n* = 370	*n* = 134	*n* = 128	*n* = 632	*n* = 184	*n* = 74	*n* = 97	*n* = 355
Range (years)	21-70	22-64	25-70	21-70	22-71	24-65	25-70	22-71
Median (IQR)	40 (31-51)	35 (28-41.25)	39.5 (32-48.75)	38 (30-49)	41.5 (31-54)	35 (28-42.25)	38 (31-48.5)	38 (30-50)
Country of birth	*n* = 371	*n* = 135	*n* = 127	*n* = 633	*n* = 182	*n* = 74	*n* = 98	*n* = 355
Australia	248 (67)	113 (84)	69 (54)	430 (68)	114 (63)	64 (87)	65 (66)	243 (69)
Other	123 (33)	22 (16)	58 (46)	203 (32)	68 (37)	10 (13)	33 (34)	112 (31)
Live with school-aged children	*n* = 372	*n* = 135	*n* = 127	*n* = 634	*n* = 182	*n* = 74	*n* = 100	*n* = 356
Yes	119 (32)	33 (24)	41 (32)	193 (30)	56 (31)	18 (24)	27 (27)	101 (28)
No	253 (68)	102 (76)	86 (68)	441 (70)	126 (69)	56 (76)	73 (73)	255 (72)
Employment status	*n* = 371	*n* = 134	*n* = 127	*n* = 632	*n* = 182	*n* = 73	*n* = 100	*n* = 355
Full-time	108 (29)	85 (63)	77 (61)	270 (43)	55 (30)	38 (52)	64 (64)	157 (44)
Part-time	232 (63)	49 (37)	50 (40)	331 (52)	112 (62)	35 (48)	36 (36)	183 (52)
Other (casual, bank, pool)	31 (8)			31 (5)	15 (8)			15 (4)
Years practised	*n* = 367	*n* = 133	*n* = 125	*n* = 625	*n* = 184	*n* = 72	*n* = 99	*n* = 355
Range (years)	0-50	0.5 – 40	0-47	0-50	0-47	1-41	1-48	0-48
Mean (SD)	16.4 (12.9)	10.7 (8.9)	16.1 (11.2)	15.1 (12.0)	18.0 (13.5)	11.8 (8.5)	15.2 (11.0)	16.0 (12.2)
Years employed at health service	*n* = 370	*n* = 134	*n* = 128	*n* = 632	*n* = 184	*n* = 73	*n* = 98	*n* = 355
Range (years)	0-45	0-25	0-28	0-45	0-39	0-30	0-31	0-39
Median (IQR)	6 (2-12)	4 (2-8)	15 (7-24.5)	5 (2-11)	6.5 (2-15)_	5 (2-9)	4 (2-11)	5 (2-12)
General health status	*n* = 358	*n* = 134	*n* = 125	*n* = 617	*n* = 184	*n* = 74	*n* = 100	*n* = 358*
Good/very good/excellent	310 (87)	120 (90)	110 (88)	540 (88)	147 (80)	62 (84)	83 (83)	292 (82)
Fair/poor/very poor	48 (13)	14 (10)	15 (12)	77 (13)	37 (20)	12 (16)	17 (17)	66 (18)
COVID-19 contact status	*n* = 343	*n* = 134	*n* = 123	*n* = 600	*n* = 174***	*n* = 74***	*n* = 98***	*n* = 346***
No direct contact	272 (79)	122 (91)	95 (77)	489 (82)	85 (49)	50 (68)	43 (44)	178 (51)
Direct contact, negative test	69 (20)	12 (9)	27 (22)	108 (18)	76 (44)	21 (28)	51 (52)	148 (43)
COVID-19 diagnosis	1 (1)	0 (0)	1 (1)	3 (1)	13 (8)	3 (4)	4 (4)	20 (6)

^¥^Owing to missing values, *n* varies for each characteristic.

*P < 0.05, **P < 0.01, ***P < 0.001, *χ*^2^ tests.

^#^Response options: No direct contact with people with known COVID-19 diagnosis, direct contact with people who have had COVID 19 diagnosis which resulted in self-isolation or testing (with a negative COVID 19 result), diagnosed with COVID 19.

In both waves, the most reported COVID-19 concerns related to infecting family with COVID-19 (52% and 53% respectively) and health of family members (53% for both waves). Overall there was a significant increase from wave 1 to wave 2 regarding concern about colleagues having COVID-19 (*P <* 0.001; Table 2). Among doctors, there was a significant increase in the proportion reporting concern about falling ill from COVID-19 (*P* < 0.05) and colleagues having COVID-19 (*P* < 0.001).

**Table 2.** Respondents’ psychosocial concerns about COVID-19 (*n* (%) extremely/very concerned)

	Wave 1				Wave 2			
	Nurses and midwives (*n* = 345-346)	Allied health staff (*n* = 134-135)	Doctors (*n* = 123)	Total (*n* = 601-603)	Nurses and midwives (*n* = 175)	Allied health staff (*n* = 74)	Doctors (*n* = 97-98)	Total (*n* = 346)
Falling ill as a result of COVID-19	101 (29)	13 (10)	12 (10)	126 (21)	59 (34)	11 (15)	21 (21)*	91 (26)
Passing COVID-19 on to family members	203 (59)	60 (45)	50 (41)	313 (52)	109 (62)	29 (39)	44 (45)	182 (53)
Your family’s health	203 (59)	63 (47)	51 (42)	317 (53)	220 (63)	31 (42)	44 (45)	185 (53)
Your colleagues having COVID-19	126 (37)	27 (20)	19 (16)	172 (29)	82 (47)*	19 (26)	35 (36)^***^	136 (39)^***^
Hospital patients having COVID-19	132 (38)	28 (21)	25 (20)	185 (31)	68 (39)	10 (14)	17 (18)	95 (28)
Caring for a patient who has or has suspected COVID-19	157 (46)	38 (28)	22 (18)	217 (36)	76 (43)	13 (18)	20 (21)	109 (32)
Falling ill as a result of COVID-19	101 (29)	13 (10)	12 (10)	126 (21)	59 (34)	11 (15)	21 (21)	91 (26)

*P < 0.05, **P < 0.01, ***P < 0.001, *χ*^2^ tests.

The most frequently reported work impacts of COVID-19 in both waves related to concerns about getting COVID-19 (82% and 84%), feeling more stress at work (62% and 70%) and cancelling annual leave (49% and 63%). Most respondents in both waves agreed that the pandemic had provided an opportunity to learn (91% and 92%) and improved their infection control knowledge (80% and 84%) (Table 3).

**Table 3.** Impact of COVID-19 on respondents’ work lives (*n* (%) strongly agree/agree)

	Wave 1				Wave 2			
	Nurses/midwives (*n* = 286-346)	Allied health (*n* = 110-135)	Doctors (*n* = 118-123)	Total (*n* = 497-600)	Nurses/midwives (*n* = 151-175)	Allied health (*n* = 64-74)	Doctors (*n* = 93-98)	Total (*n* = 296 – 344)
My job puts me at risk of getting COVID-19	282 (82)	101 (76)	110 (89)	493 (82)	146 (84)	52 (70)	92 (95)	290 (84)
I feel more stress at work	224 (65)	76 (57)	70 (57)	370 (62)	122 (71)	49 (66)	70 (72)*	241 (70)*
I have had to do work tasks that I do not usually do	167 (50)	84 (63)	63 (52)	314 (53)	117 (68)***	51 (70)	50 (52)	218 (64)^**^
I have had to do more work than I usually do	148 (43)	42 (31)	41 (34)	231 (39)	109 (64)***	37 (50)*	57 (60)^***^	203 (60)^***^
The situation has brought me closer to my manager	84 (25)	43 (32)	42 (35)	169 (28)	44 (26)	31 (42)	31 (33)	106 (32)
There is more conflict amongst colleagues at work	65 (20)	24 (18)	19 (16)	108 (18)	46 (27)	24 (33)*	26 (27)	96 (28)^***^
There is an increased sense of togetherness and cooperation among the staff	220 (64)	92 (69)	91 (75)	403 (67)	96 (56)	49 (66)	56 (58)*	201 (59)^**^
I have considered resigning because of COVID-19	46 (14)	13 (10)	10 (8)	69 (12)	39 (22)*	10 (14)	18 (19)*	67 (19)^**^
My awareness and knowledge of disease control has increased	271 (79)	105 (80)	100 (82)	476 (80)	137 (80)	67 (92)*	82 (85)	286 (84)
It has been a learning experience	301 (89)	121 (93)	112 (92)	534 (91)	151 (89)	69 (97)	87 (93)	307 (92)
I have been less busy than usual	72 (21)	57 (43)	47 (39)	176 (30)	16 (9)	9 (12)***	14 (15)***	39 (12)***
I have had to cancel or postpone my annual leave because of the COVID-19 outbreak	131 (42)	72 (57)	69 (58)	272 (49)	100 (63)***	40 (58)	61 (66)	201 (63)***
I am disappointed that I have had to cancel or postpone my annual leave due to COVID-19	111 (39)	65 (59)	63 (62)	239 (48)	75 (50)*	37 (58)	49 (61)	161 (54)
I have had to retrain or do training courses so I can do a role/job I normally would not	91 (28)	52 (40)	24 (20)	167 (29)	49 (30)	23 (33)	27 (18)	89 (27)
I do not feel very prepared to care for patients with COVID-19	87 (26)	36 (27)	16 (13)	139 (23)	39 (22)	12 (17)	14 (14)	65 (19)

*P < 0.05, **P < 0.01, ***P < 0.001, *χ*^2^ tests.

From wave 1 to wave 2, there was a significant increase in the proportion of respondents in at least one group who agreed that they: felt more stress at work, had to do new work tasks, had to do more work than usual, had considered resigning and had to cancel annual leave because of COVID-19. Significantly more respondents from at least one group agreed that there was more conflict at work than usual, and significantly fewer agreed that they experienced increased collaboration among the staff (Table 3).

For the DASS-21, Cronbach’s α in wave 1/wave 2 was 0.901/0.908 for the Depression subscale, 0.754/0.798 for anxiety and 0.886/0.883 for stress. For the total sample, all DASS-21 subscale scores were significantly higher in wave 2 than in wave 1 (Table 4).

**Table 4.** Comparison of scores on DASS-21 subscales, waves 1 and 2

		Wave 1				Wave 2			
Scale		Nurses and midwives (*n* = 391)	Allied health staff (*n* = 139)	Doctors (*n* = 138)	Total (*n* = 668)	Nurses and midwives (*n* = 184)	Allied health staff (*n* = 74)	Doctors (*n* = 100)	Total (*n* = 358)
DASS-21 Depression (range 0–21)	Mean (SD)	3.25 (4.13)	3.06 (3.32)	2.59 (3.68)	3.08 (3.87)	4.71*** (4.97)	3.59 (3.59)	3.61** (3.97)	4.17*** (4.47)
	Median (IQR)	2 (0–4)	2 (0–4)	1 (0–4)	2 (0–4)	3 (1–7)	3 (1–5.25)	2 (1–5)	3 (1–6)
DASS-21 Anxiety (range 0–21)	Mean (SD)	2.74 (3.02)	1.57 (2.05)	1.43 (2.08)	2.22 (3.20)	4.02*** (4.04)	2.70** (2.75)	2.03** (2.32)	3.20*** (3.50)
	Median (IQR)	2 (0–4)	1 (0–2)	1 (0–2)	1 (0–3)	3 (1–6)	2 (0–4)	1 (0–3)	2 (1–4)
DASS-21 Stress (range 0–21)	Mean (SD)	5.23 (4.45)	4.94 (3.65)	4.81 (3.94)	5.05 (4.16)	6.27* (4.94)	6.14* (3.92)	5.38 (3.92)	6.00** (4.48)
	Median (IQR)	5 (2–7)	5 (2–7)	4 (2–7)	5 (2–7)	6 (2–9)	6 (3–9)	5 (2–7)	6 (2–9)

*P < 0.05, **P < 0.01, ***P < 0.001, Mann-Whitney *U* tests.

For the total sample, the proportion of respondents scoring in the moderate to extremely severe range increased for depression (from 14% to 22%), anxiety (12% to 17%) and stress (14% to 20%) (not significant, Table 5).

**Table 5.** Proportion (*n* (%)) of clinicians in clinical ranges on DASS-21 subscales, waves 1 and 2

		Wave 1				Wave 2			
Scale	Ranges for clinical cut-off points[24]	Nurses and midwives (*n* = 346-353)	Allied health staff (*n* = 131-134)	Doctors (*n* = 120-125)	Total (*n* = 600-697)	Nurses and midwives (*n* = 178-180)	Allied health staff (*n* = 71-73)	Doctors (*n* = 97-98)	Total (*n* = 348-351)
DASS-21 depression (range 0–21)	Normal (0–4)	268 (78)	103 (77)	96 (77)	567 (77)	109 (61)	49 (69)	68 (69)	226 (65)
	Mild (5–6)	24 ()7	17 (13)	10 (8)	51 (8)	24 (13)	13 (18)	10 (10)	47 (14)
	Moderate (7–10)	25 (7)	8 (6)	13 (10)	46 (8)	21 (12)	5 (7)	13 (13)	39 (11)
	Severe (11–13)	12 (4)	3 (2)	3 (2)	18 (3)	11 (6)	2 (3)	5 (5)	18 (5)
	Extremely Severe (14+)	17 (5)	3 (2)	3 (2)	23 (4)	14 (8)	2 (3)	2 (2)	18 (5)
	*Moderate, severe or extremely severe (7+)*	*54 (16)*	*14 (10)*	*19 (15)*	*87 (14)*	*46 (26)*	*9 (13)*	*20 (20)*	*75 (22)*
DASS-21 anxiety (range 0–21)	Normal (0–3)	250 (71)	116 (89)	103 (84)	469 (77)	101 (57)	54 (74)	77 (79)	232 (67)
	Mild (4–5)	49 (14)	8 (6)	10 (8.)	67 (11)	34 (19)	9 (12)	14 (14)	57 (16)
	Moderate (6–7)	23 (7)	4 (3)	7 (6)	34 (6)	17 (10)	5 (7)	3 (3)	25 (7)
	Severe (8–9)	17 (5)	2 (2)	3 (2)	22 (4)	9 (5)	2 (3)	1 (1)	12 (3)
	Extremely severe (10+)	14 (4)	1 (1)	0 (0)	15 (3)	17 (10)	3 (4)	2 (2)	22 (6)
	*Moderate, severe or extremely severe (6+)*	*54 (15)*	*7 (5)*	*10 (8)*	*71 (12)*	*43 (24)*	*10 (14)*	*6 (6)*	*59 (17)*
DASS-21 stress (range 0–21)	Normal (0–3)	262 (76)	104 (78)	92 (77)	458 (76.)	124 (69)	48 (66)	75 (77)	247 (70)
	Mild (4–5)	34 (10)	12 (9)	13 (11)	59 (10)	15 (8)	8 (11)	10 (10)	33 (9)
	Moderate (6–7)	26 (8)	11 (8)	10 (8)	47 (8)	17 (9)	11 (15)	8 (8)	36 (10)
	Severe (8–9)	15 (4)	6 (5)	4 (3)	25 (4)	16 (9)	6 (8)	4 (4)	26 (7)
	Extremely severe (10+)	10 (3)	0 (0)	1 (1)	11 (2)	8 (4)	0 (0)	1 (1)	9 (3)
	*Moderate, severe or extremely severe (6+)*	*51 (15)*	*17 (13)*	*15 (13)*	*83 (14)*	*41 (23)*	*17 (23)*	*13 (13)*	*71 (20)*

In the general linear models, the main effect for wave was significant for all three subscales (Table 6). Compared with the first wave, the second wave was associated with significantly higher depression (*P* < 0.001), anxiety (*P* < 0.001) and stress (*P* < 0.01) scores. Compared with doctors, nurses/midwives had significantly higher anxiety scores (*P* < 0.001). Older age and better self-rated general health were associated with lower scores on all subscales (*P* < 0.001). Most effect sizes were small.

**Table 6.** Impact of wave (time point) and discipline group on respondents’ depression, anxiety and stress scores

Variable	Depression				Anxiety				Stress			
	B	95% CI	*F*(1, 951)	Partial *η*^2^	B	95% CI	*F*(1, 951)	Partial *η*^2^	B	95% CI	*F*(1, 951)	Partial *η*^2^
Sex												
Female	0.28	−0.46, 1.02	0.55	0.00	0.27	−0.26, 0.79	0.98	0.00	0.33	−0.45, 1.11	0.67	0.00
Male (ref)												
Age	−0.05	0.07, −0.02	17.51^***^	0.02 S	−0.06	−0.07, −0.04	54.37^***^	0.05 S	−0.06	−0.08, −0.04	27.38^***^	0.03 S
Self-rated general health^1^	−1.66	−2.03, −1.30	81.17^***^	0.08 M	−0.97	−1.22, −0.70	53.60^***^	0.05 S	−1.36	−1.75, −0.98	49.06^***^	0.05 S
Group												
Nurses and Midwives	0.56	−0.10, 1.22	2.25^2^	0.00	1.41	0.94, 1.88	28.64^2^^***^	0.04 S	0.42	−0.28, 1.12	0.93^2^	0.00
Allied Health Staff	−0.00	−0.77, 0.77		0.00	−0.06	−0.61, 0.49		0.00	0.08	−0.74, 0.89		0.00
Medical staff (ref)												
Wave												
2	0.91	0.40, 1.43	12.08^***^	0.01 S	0.97	0.60, 1.34	26.60^***^	0.03 S	0.80	0.25, 1.34	8.26^**^	0.01 S
1 (ref)												

^1^1, very poor; 2, poor; 3, fair; 4, good; 5, excellent,

^2^ *F*(2,951).

B, unstandardized coefficient; OR, odds ratio; CI, confidence intervals; *η*^2^, eta squared; ref, reference category; S, small effect size; M, medium effect size.

*P < 0.05, ^**^P < 0.01, ^***^P < 0.001.

## Discussion

This study has established that, controlling for demographic and health variables, the mental health of Australian hospital clinicians was significantly worse during the second wave of the COVID-19 pandemic compared to the first wave. The cumulative effect of respondents’ sustained increased workloads, cancellation of annual leave, and increased concern about COVID-19 infection among themselves, their families and their colleagues, could all have contributed to higher levels of psychological distress during wave 2. Notably, during both surveys respondents also reported positive learning experiences during the pandemic.

The strengths of this study include that outcomes were assessed at two distinct time points 5 months apart, which coincided with the end of two subsequent waves of the pandemic during 2020. A validated instrument, the DASS-21, was used to assess depression, anxiety and stress. To our knowledge, a limited number of studies have assessed psychological distress over multiple waves of the pandemic [9, 11]. We are not aware of any other studies that have reported clinicians’ specific COVID-19 concerns or work impacts over time.

The study has several limitations. Firstly, it was limited to a single health service which provided care for a large proportion of COVID-19-positive patients during 2020; therefore, the findings cannot be generalized to other contexts where the burden on clinical staff was perhaps less. Secondly, there were insufficient matched responses to conduct a longitudinal analysis within the same participants. Therefore, a comparison of two different samples was conducted, and it cannot be concluded that the symptoms reported by the respondents were the result of the COVID-19 pandemic. These findings should be interpreted with caution; although there were no significant differences between the two groups in terms of demographic characteristics, there are many other factors that may have contributed to these findings, including respondents’ personal circumstances and history of mental health problems; future studies should include these factors and adjust for them in analyses. Thirdly, the response rate was low, especially in the second wave; this is not unusual for unsolicited surveys among healthcare workers during infectious disease outbreaks [21]. Finally, this study did not assess other mental health problems such as insomnia, burnout and post-traumatic stress disorder which may be experienced by hospital clinicians during infectious disease outbreaks [28].

The prevalence of moderate to extremely severe depression (22%) and anxiety (17%) in our study during wave 2 are slightly less than the pooled prevalences reported in meta-analyses [1, 28]; this may be related to the relatively low number of COVID-19 cases and deaths in Australia compared with other countries [29]. Consistent with other studies, respondents’ most reported concerns were risk of contracting COVID-19 themselves and passing it on to their families [2, 4, 5, 12]. The finding that significantly more wave 2 respondents than wave 1 respondents reported increased workloads during the pandemic aligns with a study conducted in the UK [12].

Longitudinal data from existing studies [8, 9] suggest that among some healthcare workers, psychological distress may decline once the COVID-19 pandemic resolves; however, health services should consider offering ongoing occupational and psychosocial support both during and after the COVID-19 pandemic. This is especially important to buffer clinicians from the impact of poor mental health in the workplace [2, 4, 25] given the increased proportions reporting work-related conflict and considering resigning because of COVID-19, and the decreased proportion experiencing togetherness and cooperation among colleagues, in the wave 2 compared with the wave 1 survey in this study. Healthcare worker educators, working at undergraduate level and those responsible for continuous professional development, should consider preparing healthcare workers for pandemics and public health events. For example, healthcare workers would benefit from acquiring the necessary skills and knowledge to respond to fears and concerns about caring for patients during a global pandemic, including the development of self-care, resilience and healthy coping strategies [30]. Providing education and support for psychological well-being will assist health services to have an adequate, resilient and sustainable workforce.

In conclusion, this study found that psychological distress among hospital clinicians was significantly worse during the second wave of the pandemic, compared to the first wave. The specific occupational and psychosocial concerns and impacts reported by hospital clinicians in this study indicate the need for appropriate and continued psychosocial initiatives to protect the mental health of staff during continuing or new pandemics.
